# Pyrimethamine reduced tumour growth in pre-clinical cancer models: a systematic review to identify potential pre-clinical studies for subsequent human clinical trials

**DOI:** 10.1093/biomethods/bpae021

**Published:** 2024-03-29

**Authors:** Sivananthan Manoharan, Lee Ying Ying

**Affiliations:** Molecular Pathology Unit, Cancer Research Centre, Institute for Medical Research, National Institutes of Health, Ministry of Health Malaysia, Shah Alam 40170, Selangor, Malaysia; Department of Biomedical Sciences, Asia Metropolitan University, Johor Bahru 81750, Johor, Malaysia

**Keywords:** animal models, cancer, clinical trial, pyrimethamine, STAT3, systematic review

## Abstract

Pyrimethamine (PYR), a STAT3 inhibitor, has been shown to reduce tumour burden in mouse cancer models. It is unclear how much of a reduction occurred or whether the PYR dosages and route of administration used in mice were consistent with the FDA's recommendations for drug repurposing. Search engines such as ScienceDirect, PubMed/MEDLINE, and other databases, including Google Scholar, were thoroughly searched, as was the reference list. The systematic review includes fourteen (14) articles. The risk of bias (RoB) was assessed using SYRCLE's guidelines. Due to the heterogeneity of the data, no meta-analysis was performed. According to the RoB assessment, 13/14 studies fall into the moderate RoB category, with one study classified as high RoB. None adhered to the ARRIVE guideline for transparent research reporting. Oral (FDA-recommended) and non-oral routes of PYR administration were used in mice, with several studies reporting very high PYR dosages that could lead to myelosuppression, while oral PYR dosages of 30 mg/kg or less are considered safe. Direct human equivalent dose translation is probably not the best strategy for comparing whether the used PYR dosages in mice are in line with FDA-approved strength because pharmacokinetic profiles, particularly PYR's half-life (t_1/2_), between humans (t_1/2_ = 96 h) and mice (t_1/2_ = 6 h), must also be considered. Based on the presence of appropriate control and treatment groups, as well as the presence of appropriate clinically proven chemotherapy drug(s) for comparison purposes, only one study (1/14) involving liver cancer can be directed into a clinical trial. Furthermore, oesophageal cancer too can be directed into clinical trials, where the indirect effect of PYR on the NRF2 gene may suppress oesophageal cancer in patients, but this must be done with caution because PYR is an investigational drug for oesophageal cancer, and combining it with proven chemotherapy drug(s) is recommended.

## Introduction

A report in 2020 showed approximately 19.3 million new cancer cases were registered with almost 10 million cancer-associated mortality/deaths. Breast cancer has exceeded lung cancer as the most often diagnosed cancer, accounting for 2.3 million (11.7%) of new cases in 2020. Even though breast cancer is becoming more commonly diagnosed, lung cancer remains the leading cause of cancer-related death, accounting for an estimated 1.8 million (18%) cases, followed by colorectal, liver, stomach, and breast cancer. The global cancer burden is predicted to rise by 47% between 2020 and 2040 [1].

According to the National Cancer Institute [2], cancer patients have access to a variety of cancer treatment options. The cancer treatment options include chemotherapy, hormone therapy, immunotherapy, radiation therapy, photodynamic therapy, stem cell transplantation, surgery, targeted therapy, biomarker testing, and thermotherapy. Although there are several cancer treatments available, chemotherapy and radiation therapy are the most popular. More potent anticancer drugs are required as cancer resistance to chemotherapy drugs grows, limiting overall treatment success and resulting in disease relapse. The addition of a novel or new drug is proposed as one way to reduce or overcome resistance to cancer therapy [3].

Since 2016, the discovery of pyrimethamine (PYR), a STAT3 inhibitor, as an investigational anticancer drug has been widely reported. PYR is mainly used as an antiprotozoal drug to treat Plasmodium species (malaria) and other protozoan parasites. Repurposing PYR as an anticancer drug is advantageous because the drug has been extensively studied, whereas drug discovery from scratch is a complex, time-consuming, and risky process. PYR is a competitive inhibitor that inhibits the dihydrofolate reductase enzyme, which is required for DNA synthesis, affecting the regeneration of tetrahydrofolic acid [4–5]. STAT3 is an important mediator that controls the cancer environment and promotes cancer development, as well as a favourable target for the anticancer immune response [6]. Although several publications have reported PYR's ability to suppress tumours in mice and preclinical models, it is unclear to what extent the suppression occurred or whether the PYR dosages used in mice are consistent with FDA-approved doses, making clinical translation easier. In general, for the anti-parasitic activity, the FDA approved a 50 mg/day PYR tablet via oral route for a maximum of 8 weeks of use in humans (without considering a chronic maintenance period) [7]. Brown et al. [8–9] conducted a leukaemia clinical trial and reported that the patient had stable disease after taking 50 mg/day for 12 months. In this systematic review, the potency of PYR in suppressing tumours in mouse models, as well as the dosages involved, were thoroughly examined and analysed. To the authors' knowledge, this is the first systematic review of PYR efficacy in tumour suppression in various preclinical cancer models. The first author (SM) of this article has 4 years of direct hands-on experience dealing with different formulations of PYR for tumour suppression in both male and female NOD-SCID-Gamma (NSG) and female nude mice-induced cancer models in an animal biosafety level 2 (ABSL2) facility at the Institute for Medical Research, National Institutes of Health, Ministry of Health, Malaysia, following the ARRIVE guidelines.

## Materials and methods

The current systematic review used the Preferred Reporting Items for Systematic Reviews and Meta-Analyses (PRISMA) guideline to develop the manuscript. The current review's protocol was not prepared or registered. The flow diagram was created using Review Manager 5.4.1 software, a Cochrane review software [10].

### Research questions

The current systematic review addresses five critical questions, which are as follows:

1. What is the effectiveness of PYR in suppressing tumours in cancer-induced mouse models?
2. Were the PYR dosages in mice within the FDA-approved range for antiparasitic activity?
3. Are there any differences in PYR pharmacokinetic profiles between humans and mice that could affect the direct translation of pre-clinical research into human clinical trials?
4. Is there an additional treatment group included to compare the efficacy of PYR to the standard chemotherapy drug in the PYR cancer study?
5. Which studies show high potential for clinical trials?

### Search strategies, article eligibility criteria, and data charting procedures

The crude articles were thoroughly searched on ScienceDirect (n = 74), PubMed/MEDLINE (n = 167), and Google Scholar (n = 183). The keywords used were ‘pyrimethamine cancer’, ‘pyrimethamine oncology’, and ‘pyrimethamine tumour growth’, with a search period spanning 2005 to October 2023. The articles were independently screened, and those related to non-animal studies (non-animal works and PYR subtypes or derivatives) were excluded from the primary analysis. The reference list of the selected articles was carefully reviewed for the inclusion of any additional articles (n = 1). In this systematic review, two authors worked independently to chart the data, including screening the titles, abstracts, and text. The search was repeated two months later (on December 21, 2023), but no new articles were found.

The article selection criteria include:

1. in vivo/animal cancer models
2. PYR group as the intervention
3. proper controls
4. reported tumour suppression efficacy (in mm^3^ or any unit easily converted to mm^3^)
5. English-only literature published between 2005 and December 2023.

### Risk of bias assessment (RoB)

The RoB was assessed following SYRCLE's guidelines [43]. This guideline was developed specifically to assess the risk of bias in animal studies. In this assessment, the included articles were checked for ten main criteria, known as:

1. sequence generation
2. baseline characteristics
3. allocation concealment
4. random housing
5. blinding (performance)
6. random outcome assessment
7. blinding (detection)
8. incomplete outcome data
9. selective outcome reporting, and
10. Other sources of bias

### Mouse-to-human dose translation

According to a Nature Metabolism article [42], we calculated the dose translation using the described method. The article states that one can calculate the human dose (mg/kg) using the following formula:

$$\text{Human dose}\;\left(\text{mg}/\text{kg}\right)=\text{Animal dose}\;\left(\text{mg}/\text{kg}\right)\;\times \;\frac{\text{Animal Km}\;\left(3\;\text{kg}/m^{2}\right)}{\text{Human Km}\;\left(37\;\text{kg}/m^{2}\right)}$$

The researchers will multiply the PYR dose used in an animal study, which was 20 mg/kg, by 0.08 (3 kg/m^2^/37 kg/m^2^) to calculate a human dose of 1.6 mg/kg. According to the same article in Nature Metabolism, the average adult human weight is 60 kg. Later, the 1.6 multiplied by 60 yields 96 mg PYR of adult dose. The 96 mg human PYR dose is equivalent to 20 mg/kg PYR in the mouse study. Animal doses can be calculated using the same formula in the presence of human dosages.

## Results and discussion

### Study inclusion

**Figure 1.**
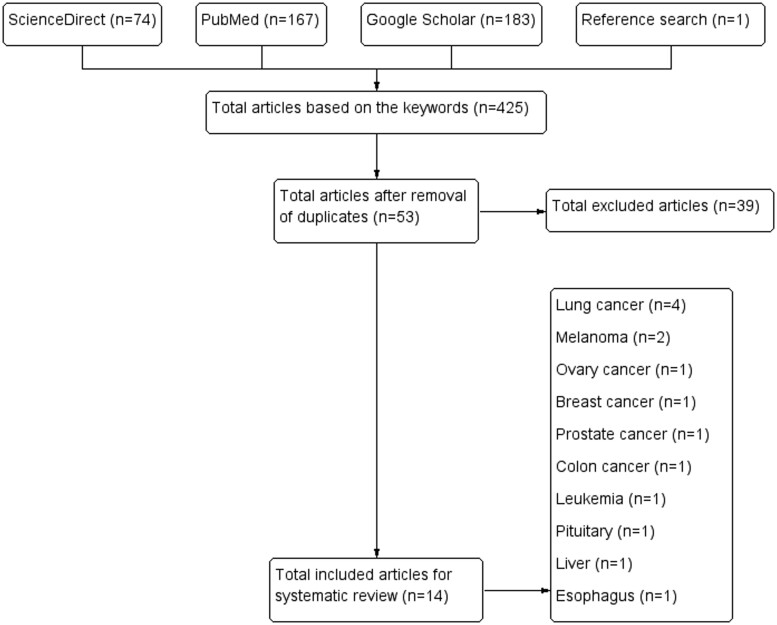
Flow chart describing the inclusion and exclusion of articles for systematic review.

This review includes 14 articles that met the inclusion criteria for a systematic review among 425 pieces of crude literature. Figure 1 depicts the entire process of literature inclusion for this study. Although a few have previously reported on this topic, it has resurfaced in the last 7 years, with one clinical trial in leukaemia patients [11]. In 2011, researchers demonstrated PYR's STAT3 inhibitory function by observing its ability to inhibit STAT3 in polycystic kidney disease models. However, researchers did not discover the STAT3 inhibitory effect of PYR in cancer until 2018 [12]. Huang et al. [12] stated that Khan et al. [13] were the first to show the STAT3 inhibitory effect of PYR in breast cancer. In vitro models showed that STAT3 inhibition by PYR suppressed breast cancer cell growth and invasion.

STAT3 belongs to the STAT family of cytoplasmic transcription factors. STAT3 is transiently activated and tightly regulated in the normal physiological state [11, 14]. STAT3 activation is associated with a poor prognosis in patients suffering from lung cancer, liver cancer, renal cell carcinoma, gliomas, prostate, breast, and colon cancer [14, 15]. Targeting the STAT3 signalling pathway has emerged as a promising therapeutic approach for many cancers [16]. Additionally, inhibiting STAT3 has been shown to produce immunoregulatory effects. Encouraging exocytosis increases CD8^+^ T-cell cytotoxicity [12]. Additionally, STAT3 plays an important role in tumour progression and metastasis. It regulates cellular proliferation, invasion, migration, and angiogenesis, all of which are critical for cancer metastasis [14]. Based on Fig. 1, it is quite evident that PYR is gaining popularity among researchers as they seek to learn more about its anticancer properties.

### Characteristics of the included articles

Table 1 lists 14 articles for qualitative analysis. There are ten types of cancer: lung, leukaemia, ovary, prostate, colon, pituitary, liver, melanoma, oesophagus, and breast. Four articles discuss the tumour suppression effect of PYR in lung cancer models. The mice were between four and eight weeks old. Different types of intervention were carried out, with the lowest concentrations of PYR being 100 μg and 500 μg injected into the animals, but the authors did not specify the type of injection used, whether intravenous (IV), intramuscular (IM), intraperitoneal (IP), etc. Interestingly, while others used female mice, only a few articles mentioned the use of male mice. Studies have shown that female mice have more drastic effects than male mice after treatment, which is why they are most likely used for animal experiments [18]. However, it is encouraged to use both sexes and not limit the experiments to one sex. Comparing research outcomes for tumour suppression in both sexes would provide additional information and strengthen research efforts except for gender-specific cancers like breast and ovarian cancers where only female mice are needed instead of using both sexes. Besides, most of the mice cancer models used in Table 1 were transplantable which can be immunologically different from the primary transgenic model. It is understood that the procedure of producing and characterizing transgenic mice takes several years. Moreover, the procedure to produce transgenic mice is labour intensive and needs considerable funding [45]. Probably, these could explain why most of the mice models used in table 1 are transplantable which is easy to be carried out and widely used in cancer research especially in subcutaneous tumour cells/fragment inoculation procedure.

**Table 1.** Characteristics of the included articles.

Study ID	Mice species/population	Sex	Age (week)	*n*/group	Type of cancer	Name of cell line/xenograft	Cell number/fragment and site of inoculation	Intervention & comparator (mainly vehicle control)	Human equivalent dose (mg/day) FDA-approved dose for 60 kg adult: 50-75 mg/d	Possibility to repurpose the drug	Outcome (PYR vs Control) Mean tumour shrinkage (mm^3^)	Mean tumour weight (g)
**Khan [13]**	i) BALB/c ii) BALB/c-NeuT transgenic mice	F	6	i) 14 ii) 14	Breast	TUBO	1 × 10^7^, MFP	i) Pyr (60 mg/kg), p.o, OD, 41 days ii) Pyr (60 mg/kg), p.o, OD, 48 days	288	NO	NA (Contacted author but no reply)	NA
**Kim [26]**	BALB/cSLC-nu/nu	F	7	5	Lung	H460	1 × 10^7^, s.c	Pyr (20 mg/kg), i.p, 5x/week, 2 weeks	96	NO (FDA indication: only oral route)	650 vs 1600	0.38 vs 0.85
**Lapidot [21]**	NSG	F	6	8	Lung	MS4	∼3 mm^3^, s.c	Pyr (75 mg/kg), p.o, OD, 22 days	360	NO	810 vs 950	NA
**Lin [27]**	BALB/c nu/nu mice	F	6	6	Lung	A549	1 × 10^7^, s.c	Pyr (30 mg/kg), i.p, OD, 3x/week, 25 days	144	NO (FDA indication: only oral route)	2000 vs 4500	2.1 vs 5.8
**Liu [17]**	C57BL/6J	M	5-6	5	Lung	Lewis	1 × 10^7^, s.c	Pyr (7.5 or 15 mg/kg), p.o, OD, 2 weeks	36 or 72	YES (but Lewis is a murine cancer cell)	3760 & 1640 vs 6700	NA
**Liu [4]**	BALB/c-nude	F	5-6	5	Ovary	SKOV3 & A2780	5.0 × 10^6^, i.p	Pyr (100 μg & 500 μg), ijc 4x/week, 60 days	NA	NA	NA (tumour located in peritoneum)	NA
**Sharma [28]**	NOD/SCID	F	6-8	5	Leukemia	HL60 & THP1	2 × 10^6^, s.c	Pyr (25 mg/kg), p.o, OD, 18 days	120	MAY BE (if well-tolerated in future phase 1 study)	1400 vs 2800 1100 vs 2400	NA
**Zhou [5]**	Nude	NA	4-5	8	Prostate	PC3	6 × 10^6^, s.c	Pyr (15 mg/kg), p.o, OD, 30 days	72	YES	500 vs 1700	0.27 vs 1.0
**Dong [29]**	BALB/c	M	5	4	Colon	CT26	1 × 10^5^, s.c	Pyr (60 mg/kg), i.g, OD, 14 days	288	NO	150 vs 900	0.15 vs 0.82
**Dai [30]**	NOD-SCID	F	6	5	Pituitary	GH3	2 × 10^6^, s.c	Pyr (30 mg/kg), p.o, 5 days/week, 8 weeks	144	MAY BE (but GH3 is a murine cancer cell)	1900 vs 4800	NA
**Giammarioli [31]**	CB.17 SCID/SCID	F	4-5	8	Melanoma	501	2 × 10^6^, s.c	Pyr (30 or 60 mg/kg), p.o, 5 days/week, 30 days	144 or 288	NO (no effect at 30 mg/kg)	NA	0.3 (control) vs 0.3 (30 mg/kg) & 0.16 (60 mg/kg)
**Tommasino [32]**	CB.17 SCID/SCID	F	4-5	8	Melanoma	501	2 × 10^6^, s.c	Pyr (45 mg/kg), p.o, 5 days/week, 35 days	216	NO	650 vs 1100	NA
**Wang [33]**	Nude	M	4	5	Liver	SMMC-7721 & SMMC-7721(shCon) & SMMC-7721 (shBNIP3)	4 × 10^6^, s.c	Pyr (60 mg/kg), p.o, OD, 3 weeks & Pyr (30 mg/kg), p.o, OD, 3 weeks	144 or 288	MAY BE for 30 mg/kg	550 vs 1000 (shCon-60 mg/kg) 800 vs 1300 (shBNIP3-60 mg/kg) 1150 vs 1500 (SMMC-7721-30 mg/kg)	0.12 vs 0.28 (shCon) 0.2 vs 0.35 (shBNIP3) 0.3 vs 0.55 (SMMC-7721)
**Paiboonrungruang [34]**	Sox2CreER; LSL-Nrf2^E79Q/+^	NA	NA	4	Esofagus	NA (genetic crossed)	NA (genetic crossed between 2 strains)	Pyr (30 mg/kg), p.o, OD, 5 weeks (Note: in combination with other drug/s)	144	MAY BE	NA	′NA

p.o: oral; OD: once daily; i.p: intraperitoneal; s.c: subcutaneous; *n*: number; F: female; M: male; NA: not available; MFP: mammary fat pad; i.g: intra-gastric; Ijc: injection.

The mouse-to-human dose translation [42] and the possibility of repurposing PYR in Table 1 did not account for the pharmacokinetic parameters, particularly the half-life of PYR in mice (t_1/2_ = 6 h) and humans (t_1/2_ = 96 h). The authors discussed this limitation and potential solutions for translating in vitro and in vivo data to human clinical trials in the limitation section of the current work, which is located above the conclusion.

However, an intriguing point was discovered by the authors in the information presented in Table 1: the PYR dosage used in the animal study. Only Liu et al. [17] used 7.5 mg/kg PYR administered orally, which, when converted to a human dose, is less than 50 mg/day and could be repurposed directly into a Phase 2 human clinical trial for long-term study. But it is worth noting that the authors used immunocompetent C57BL/6J mice. Originally developed for use in cancer research and immune responses, C57BL/6 mice are well known for their immunogenicity. It is well known that natural killer cell activity, which contributes significantly to antitumor activity, is higher in C57BL/6 mice than in any other mice [19]. To the best of the authors’ literature search, no direct experimental evidence shows that PYR can increase the activity of natural killer cells and subsequently lead to a reduction in tumour size/volume. It has been shown that human natural killer cell cytotoxicity against K-562, which is the cell derived from leukaemia patient was reported to be markedly weakened by PYR in vitro [46]. Furthermore, Lewis cells are derived from mouse lung cells rather than human cells [41] and this could explain why the tumour can be formed in immunocompetent mice without tumour rejection. Several other articles have also used animal cancer cell lines such as TUBO, CT26, and GH3 for PYR drug testing experiments. Although this practice is acceptable for experimental work, it is preferable to use human-related cancer cells because they are easier to interpret and identify potential opportunities for translating the results into clinical trials. Two studies used 15 mg/kg of PYR orally in mice, one of which was conducted by the same authors as previously discussed [5, 17]. This dose, when converted, is equivalent to 72 mg/day in humans weighing 60 kg. These studies may also enter Phase 2 trials, with a maximum PYR human dosage of 75 mg/day for a maximum duration of 8 weeks, after which the dosage must be reduced in half beginning week 4 [7]. Other treatment durations, such as 6 months for chronic maintenance with PYR 50 mg/day, must meet certain important criteria. This is primarily for ongoing antiparasitic therapy in HIV-infected patients with CD4 counts <200 cells/mm^3^ [7]. The dosages are based on FDA-approved human antiparasitic activity. The other PYR dosages used in mouse cancer models above 15 mg/kg must be evaluated for safety in Phase 1 human clinical trials before beginning a trial on cancer patients. The other studies with a higher chance of being included in future Phase I or Phase II clinical trials are those with 25 mg/kg and 30 mg/kg PYR doses. Although the dose conversion in human subjects is 120 and 144 mg, respectively, which is far from the FDA's allowed concentration in humans, we believe it is highly likely that these studies will progress to future clinical trials. To answer this, in the limitations section of this manuscript, we go into detail about PYR's half-life in humans and mice. Khan et al. [13], Liu et al. [4], and Paiboonrungruang et al. [34] did not report PYR tumour suppression effects in mm^3^, and there is no other way to convert the data to mm^3^. Because of the limited number of articles shortlisted, we included these three articles in Table 1 to outline the study designs, types of cancers, and interventions used in PYR studies.

Although several studies are proposed for Phase I and II clinical trials, it is critical to protect patients' rights to receive investigational drugs (PYR) in conjunction with clinically proven chemotherapy. Only one article in this systematic review included sorafenib, a positive control known for treating advanced hepatocellular carcinoma [33]. This is an excellent strategy that is underutilized or ignored because it allows the effectiveness of PYR and Sorafenib to be observed in pre-clinical studies. Furthermore, this strategy will expedite the drug discovery process because these data are likely to be required in certain countries before moving forward with clinical trials. The primary goals are to assess (i) the safety of the investigational drug in combination with clinically proven chemotherapy and (ii) ensure that the addition of the investigational drug does not reduce the efficacy of the clinically proven chemotherapy. Another possibility for a clinical trial is to observe the effect of PYR on specific genes such as STAT3 and NRF2, with the hope that inhibiting these genes will result in positive outcomes in cancer patients. Simply receiving PYR and looking for gene inhibition is extremely risky for cancer patients because the observations made in preclinical studies may not be translated into patients, and in the worst-case scenario, the disease will progress to the next stage. Furthermore, it is advisable to combine the investigational drug with clinically proven chemotherapy.

### Risk of bias assessment and ARRIVE guideline for pre-clinical study

**Figure 2.**
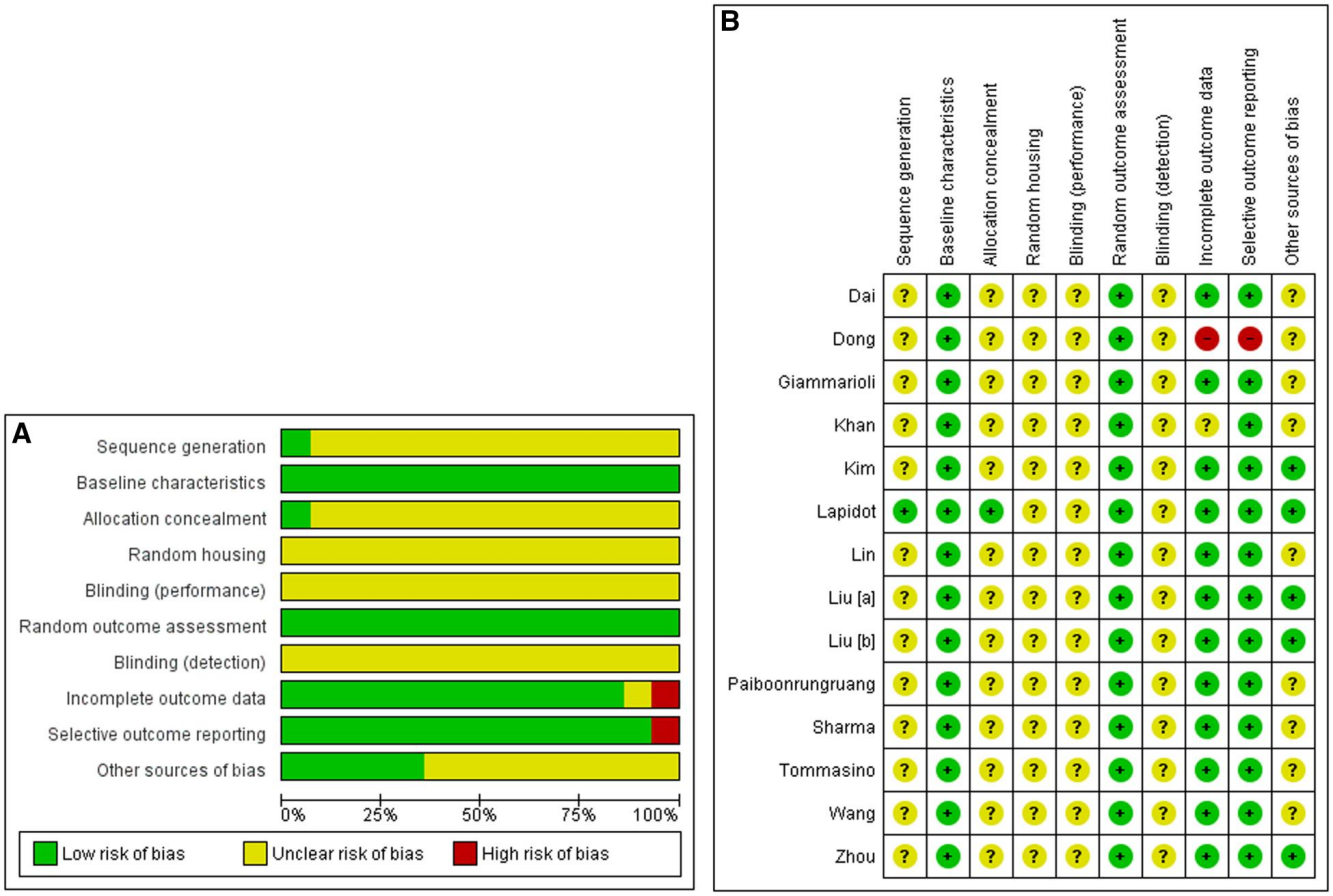
Risk of bias assessment based on SYRCLE’s guideline. The SYRCLE is a guideline to assess RoB which is related to animal studies. It is different from the Newcastle–Ottawa scale (NOS) and Cochrane’s RoB which are used to assess human-related studies for non-randomised and randomized controlled trials (RCTs), respectively [22–23, 48–49].

Figure 2 shows the RoB. According to Fig. 2, only one article used proper animal randomization. Although a few papers mentioned that the animals were randomised, none described the method used for randomization. The simplest method of randomization, such as a coin toss, is not appropriate or scientific. Instead, researchers should use a sequence generated by the software. We are unsure about the blinding steps and random housing in the reported works because no one mentioned these methods. It is unclear whether these steps were carried out. The increased manpower required when using the blinding method from the start to the end of the animal studies is likely the reason for not carrying out these steps. Some research teams are small, making blinding strategies difficult to implement. To overcome the men's power issue, we believe that at least one blinding step is required to reduce performance and detection bias. For example, the person harvesting the tumour should be unaware of the mice's grouping. Another blinding strategy is to have a different person administer the drug to the mice while another person measures tumour size and body weight. According to the Animal Research: Reporting of In Vivo Experiments (ARRIVE) guideline 2.0 [20], it is critical to follow all the parameters specified in the guideline to produce transparent reporting in animal studies. Over a thousand life science journals have endorsed the ARRIVE guideline for transparent reporting in animal research. However, according to PLOS Biology [20], recent studies have revealed that important parameters established in the ARRIVE guidelines remain absent from most published articles. This includes information on randomization (described in only 30%–40% of published articles), blinding (reported in approximately 20% of published manuscripts), sample size explanation (stated in no more than 10% of publications), and animal characteristics. Figure 2 demonstrates that none of the articles reported on blinding strategies. The first author (SM) of this manuscript conducts cancer research in mouse models following the full ARRIVE guidelines and has found it difficult or challenging to implement all parameters at times due to manpower issues. Researcher A will use software to generate sequences and then pass them on to Researcher B. Researcher B will conduct randomization, including random housing. Researcher C will measure body weight (real-time body weight is required before drug administration) and administer the drug to the mice. Researcher D will measure tumour size and body weight. Tumour harvesting and final tumour weight measurement will be performed by researcher E. Researcher F will analyse the data. A general staff is responsible for the mice's husbandry, food, and drinks. These strategies present significant manpower challenges. It is nearly impossible to carry out with a small research team of only one to three researchers. That is why, as previously suggested, at least one blinding strategy is likely required for a team of a small number of researchers. On the other hand, two articles did not mention the sex of the mice used [5, 34], and one study did not mention the age of the mice [34], which can have an indirect impact on the overall assessment of baseline characteristics, but we did not choose to devalue these articles based on these parameters because many other important parameters related to baseline characteristics are well described. Figure 2 shows that most of the included articles have a moderate risk of bias. Therefore, careful interpretation is necessary, and the results are of moderate quality. However, Dong et al. [29] is regarded as a high-risk article because the authors used different cell lines for in vitro studies but used an unrelated cell line in vivo, even though all cell lines are associated with colon cancer. Furthermore, the authors used mouse-derived CT26 cells for in vivo experiments. However, the authors did not report any related in vitro results, despite mentioning CT26 cells for in vitro work. There is no research work on translating from in vitro to in vivo.

When comparing individual articles, only Lapidot et al. [21] met many of the parameters outlined in the SYRCLE guidelines. Table 1 shows that only Lapidot et al. [21] used NSG mice, which are among the most immunocompromised mice reported to date. In general, this strain of mice has no immune system except for neutrophils and monocytes, necessitating strict and safe housing to avoid infection [24]. One possible reason the other authors did not use NSG mice but instead used more immunocompetent mice is that PYR is an immunomodulator [5] that requires immune cells to exert its activity. Immune cell is absent in NSG mice, but this environment has made NSG an excellent strain for developing tumours with a high success rate due to a low rejection rate [24, 25]. Furthermore, Lapidot et al. [21] used fragment inoculation rather than single-cell suspension. The fragment contains a mixture of cancer and mouse cells. Based on the first author's (SM) experience, the author performed tumour digestion and depletion and discovered that the patient/cell-derived xenografts (PDX/CDX) harvested from the mouse were heavily contaminated with mouse cells. The fragment inoculated into the mouse may not contain all cancer cells. The fragment could contain mouse cells, and they are likely to be in large numbers. Furthermore, the number of human cancer cells in the fragmented (3x3x3mm) PDXs used for mouse inoculation may vary. In contrast, the single-cell suspension contains human cancer cells. These may explain why, even at 75 mg/kg PYR, the tumour size differed only slightly from the control for Lapidot et al. [21]. However, 75 mg/kg of PYR in mice is too high and can cause myelosuppression. Mice developed myelosuppression after being given 60 mg/kg of PYR [34].

### Drug dosing and route of administration

PYR is an oral drug, which means it must be taken through the mouth. In mice, a dosage of 30 mg/kg is much safer. Table 1 demonstrates the administration of PYR to mice through two routes: oral and IP. Researchers primarily use the IP route in research to mimic IV drug administration while also allowing for easy drug administration. Administering the drug through the mouse tail vein is more difficult and takes more time and resources than using the IP route. Furthermore, the peritoneum is an excellent portal of entry into systemic circulation for compounds administered via IP. However, the IP route and the IV route may not be the same. After administering the drug into the mouse's peritoneal cavity in IP, the drug (a small compound) undergoes absorption and transportation to the liver via the hepatic portal vein, where it undergoes first-pass metabolism, resulting in only a small number of drugs entering the systemic circulation. This step is absent from the IV route of drug administration. This makes the IP route like an oral route. Macromolecules, on the other hand, can bypass first-pass metabolism by entering the systemic circulation through lymphatic vessels or experiencing minimal effects. The IP route allows for administering a large volume of drug, reducing stress on the animal, and eliminating the need for the drug to pass through a complex gastrointestinal tract, which could cause degradation. However, clinics rarely utilise the IP route, leading to frequent questioning and discouragement. The IP route of drug delivery in rodent studies is a good way to do preliminary pharmacological and proof-of-concept work where the goal is to see how the drug works. However, it is not a good way to do studies that want to find the main pharmacological effect, which includes pharmacokinetics for clinical translation [35]. Furthermore, administering the drug intravenously in a clinical setting may require a preclinical study involving IV administration before the clinical trial. Special care should be taken when suggesting the IV route of drug administration, as data obtained from animal studies using IP administration for human-related clinical trials needs to be interpreted cautiously. This applies to drug- and cell-based therapies [36]. According to Science in Poland [37], a preclinical trial testing PYR (GLG-805) for IV administration was going on at the Institute of Industrial Organic Chemistry in Pszczyna, Poland, in 2018. The goal was to make it easier for PYR to get to tissues. There were no further updates found for GLG-805. In 2000, researchers conducted a study where they tested PYR (1 mg/kg) via IV infusion over 2 h in 8 healthy male volunteers. The study was done to find a safe and effective way to give pyrimethamine through an IV because patients with AIDS and toxoplasmic encephallitis have low plasma PYR concentrations when they are given oral PYR therapy. Researchers prepared and dispensed a neutral, aqueous, and sterile PYR solution in sealed glass ampoules. All participants reported a high tolerance for PYR [38]. Based on the findings of Almond et al. [38], it is possible to develop an IV formulation for PYR with an appropriate, safe dosage for IV use.

### Pharmacokinetics and study limitations

There is a major limitation in the current systematic review. The plasma pharmacokinetics of PYR in mice and humans differ, particularly in terms of the drug's half-life. In mice, PYR has a half-life of 5-6 h (h), while in humans it is 96 h [39, 40]. PYR reduces the plasma concentration by half every 6 h in mice, while it takes 96 h in humans. The FDA has approved PYR for once-daily dosing. Thus, when translating animal PYR works to human clinical trials, a dose reduction in humans from animal works is likely required to avoid accumulating toxic effects in the patients' bodies. Furthermore, the high dosages used in animal studies may be attributed to filling the 4X gaps in half-life in mice (6 h + 6 h + 6 h + 6 h = 24 h) before administering the next dose (once a day). For example, if the IC_50_ for cancer cell line X was 2 µM and 50 mg/kg PYR given through the IP route in mice produced a plasma concentration maximum (Cmax) of 35.70 µM [39], then the Cmax will be halved every 6 h (35.70 µM_0-6h_ to 17.85 µM_7-12h_ to 8.925 µM_13-18h_ to 4.46 µM_19-24h_). After 24 h, PYR concentrations in the mouse's plasma remained above 2 µM. PYR is expected to reduce tumour size by at least 50%, depending on the mouse strain. This is probably not possible in NSG mice due to a lack of vital immune cells. Despite conducting a thorough search, the authors could not find an article on PYR given orally alone and the related pharmacokinetics in mice. The IP route [39] is the closest. As previously discussed, unlike macromolecules, which enter systemic circulation via lymphatic vessels, the IP route for small drugs mimics the oral route (except for passing through the gastrointestinal tract), in which the drug is absorbed and transported to the liver via the hepatic portal system, where it undergoes first-pass metabolism before entering the systemic circulation in a small amount of PYRs. A good understanding of pharmacokinetics is required to successfully translate animal studies into human clinical trials. Furthermore, the researchers must compare the in vitro IC_50_ values of PYR for the respective cell lines, or PDXs, as well as the drug concentration required to act against specific cancer markers (e.g., STAT3) in a Western Blot (WB) study. For example, in WB, if 5 µM was needed to reduce STAT3 in cancer cell line X, based on the discussion made above for IC_50_, drug half-life, and Cmax, PYR can reduce STAT3 in cell line X inoculated mice model, but it will take some time, probably more than observed in in vitro studies because the effect of the drug on 2D monolayer cells and a fully formed round or spheroid tumour in mice or humans are two different settings. Comparing these invaluable in vitro data to the half-life and Cmax of PYR in both mouse and human species (50 mg/day PYR produced 6 µM-10 µM Cmax in leukaemia patients [8, 9]), it is probably possible to successfully translate preclinical research into clinical trials. The FDA-recommended dose and route of administration should be used for repurposing PYR as a drug. Human dose reduction should follow FDA recommendations. Furthermore, a lower dose (50 mg/kg) should produce a considerable Cmax in human plasma, ensuring successful clinical work. This could explain why Brown et al. [8, 9] saw some effects of 50 mg/day PYR in leukaemia patients, whereas Sharma et al. [28] needed 25 mg/kg in mice (120 mg/day in humans) to see the effect in leukaemia preclinical models. We believe that the differences in pharmacokinetic profiles, particularly Cmax and drug half-life, play significant roles in these two species. PYR has been shown to have several other mechanisms of action in producing anti-cancer effects in different types of cancers. The detailed mechanisms of action and existing PYR clinical trial data in leukaemia patients have been discussed by Ramchandani et al. [44]. Despite several other reported mechanisms other than STAT3, it is important to look at the PYR dosages used in the WB study to identify this mechanism of action. Based on the pharmacokinetic discussions made above, any used dosages beyond 10 µM probably not reflect the actual scenarios in human subjects although they yielded good results in vitro/in vivo. It is always important to compare the results/outcomes and the pharmacokinetics information between these 2 species (human/mouse) before deriving any conclusion/s. Unlike this systematic review, Ramchandani et al. [44] mainly summarize the overall effect of PYR in different types of cancers and do not give a lead for clinical translation. Based on our discussions, since FDA-approved dose of 50 mg/day PYR in humans produced only 6 µM-10 µM Cmax in leukaemia patients, it is recommended for in vitro (2D/3D cell culture) studies including WB to use dosages less than or maximum of 10 µM because the goal of basic research is to be translated in human clinical studies and the 2D/3D research settings should be as close as possible to the clinical settings. Besides, combining PYR with proven chemotherapy drug/s for in vitro studies would be an added advantage and highly recommended. This recommendation is not just limited to PYR but also can be applied to other drug-repurposing candidates.

In this systematic review, we only considered PYR pharmacokinetic profiles established in cancer patients [8, 9]. This is because although there is some pharmacokinetic information available in regard to the treatment of PYR in patients with malaria and the PYR pharmacokinetics in pregnant women from several African countries, the pharmacokinetics of the drug (not limited to PYR alone) vary depending on the disease, race, gender, healthy versus diseased participants, nutrition, and other factors. Since Brown et al. [8, 9] already established PYR pharmacokinetics in leukaemia cancer patients in 2018, we followed these values and had discussions. Although leukaemia is a blood cancer and not a solid cancer, the pharmacokinetic values obtained from leukaemia patients are the closest we can get for cancer-related discussions. Indeed, a new set of PYR pharmacokinetics for solid cancers is required, as well as comparisons between the pharmacokinetic profiles of leukaemia and solid cancers. Currently, one active PYR-related clinical trial designated as an early phase I trial for head and neck squamous cell carcinoma with identification number NCT05678348 is found on the ClinicalTrial.gov website, but the study is still in patient-recruiting stage. This is the first PYR study for solid cancer in humans. In this open labelled single group/arm study, the 50 mg/day PYR will be given orally to the patients for 14 days with the final dose the day before the surgery [47]. The study settings are different from the leukaemia clinical trial (NCT01066663) and a direct comparison cannot be made. The second (minor) limitation is that only borderline positive to positive results for PYR tumour suppression have been reported. The search yielded no negative results in terms of tumour suppression. It is common practice not to report negative findings, which may limit the overall effectiveness of PYR. Based on the experience, the first author is convinced that PYR may have produced negative results in certain cancer cell lines or xenografts. Reporting such negative results is critical for fully understanding the effect of PYR in suppressing tumour cell lines and xenografts, particularly those from the same type of cancer.

## Conclusion

In this systematic review, we critically and thoroughly addressed all five questions:

### What is the effectiveness of PYR in suppressing tumours in cancer-induced mouse models?

Although PYR was shown to reduce tumour burden in pre-clinical models (Table 1), the findings are inconclusive because no negative results were presented. Several studies did not use the FDA's recommended route of administration. Only a few studies followed FDA regulations. Few studies have investigated the efficacy of mouse-derived cancer cells rather than human cancer cells. Moreover, based on the first author’s experience with PYR in mouse cancer models, PYR produced negative results. One of the experiences was that certain tumours that did not respond in NSG mice responded in nude mice, most likely due to the immunomodulatory effect of PYR. Some tumours have not responded in both mouse strains.

### Were the PYR dosages in mice within the FDA-approved range for antiparasitic activity?

Direct human equivalent dose translation is probably not the best strategy for determining whether the PYR dosages used in mice are consistent with the FDA-approved strength. Although few studies reported dosages within FDA-approved strength, we believe any dosages used at 30 mg/kg or lower could be considered for human studies. Sharma et al. [28] have proven this. Furthermore, the differences in pharmacokinetic profiles between humans and mice must be considered.

### Are there any differences in PYR pharmacokinetic profiles between humans and mice that could affect the direct translation of pre-clinical research into human clinical trials?

Yes. Details are discussed above. This may explain why certain studies, despite producing positive in vitro results, failed to translate into animal and human models. Many parameters, including a drug's Cmax, half-life, volume of distribution, protein binding capacity, and several others, influence the efficacy of PYR. In vitro studies should focus on dosages that can be sustained in human plasma for an extended period rather than relying on the compound's IC_50_. For example, if PYR produced an IC_50_ of 30 µM after 72 h of incubation, it is advisable to avoid using 30 µM for further experiments because, at 50 mg/kg PYR, only 6 µM-10 µM of PYR can be achieved in human plasma [8, 9]. Although 30 µM is still achievable in mouse models, working towards a human dose is recommended because the work needs to be translated into humans. Future PYR and non-PYR drug repurposing preclinical studies should focus on FDA and ARRIVE guidelines, established pharmacokinetic profiles, use of human cancer-related cell lines, combining drugs with standard chemotherapy drug(s), and dosages of in vitro testing, including WBs should be designed based on the human plasma concentration of established drug(s) rather than on an impractical high inhibitory concentration 50 (IC_50_).

### Is there an additional treatment group included to compare the efficacy of PYR to the standard chemotherapy drug in the PYR cancer study?

Yes, but only in one article. One group combined PYR with sorafenib, while three other groups tested only sorafenib (15 mg/kg), PYR (30 mg/kg), and a control. The combination drug treatment group produced only about 100 mm^3^ of tumour size at the end of the study in mice, whereas the control, PYR, and sorafenib produced around 1500 mm^3^, 1000 mm^3^, and 550 mm^3^, respectively. This demonstrates a clear synergy between the PYR and sorafenib combination. Sorafenib was used within the prescribed human dose. Furthermore, we did not observe any toxic effects in terms of significant body weight loss. We determined that this was the best way to proceed with a clinical trial. Although the study design was considered the best design in comparison with the other shortlisted articles, this design cannot be generalised for all cancers as the best way to proceed with a clinical trial. Discussions with respective stakeholders before starting preclinical work would be another best strategy for a smooth clinical translation.

### Which studies show high potential for clinical trials?

Promising drugs including for drug repurposing are usually dropped from further investigation/s due to insufficient effectiveness of the drug or superiority to alternate treatments [50]. In this systematic review, we thoroughly examined the methodologies used in the shortlisted evidence/articles to give a clear lead article for human clinical trials. This is because we believe before concluding the insufficient efficacy of the repurposed drug, the used methodologies need to be analysed thoroughly. According to Table 1, the following studies can be considered for future clinical trials: Zhou et al. [5], Liu et al. [17], Sharma et al. [28], Wang et al. [33], and Paiboonrungruang et al. [34]. Human-related cancer cells were used in all studies except for Paiboonrungruang et al. [34] and Liu et al. [17], where genetic crosses between mouse strains were used to develop the model and Lewis lung cancer cells, which are of mouse origin, were used, respectively, but the work designs and the used PYR drug concentrations (7.5 mg/kg to 30 mg/kg) in both articles justify the works being included for future clinical trials. Although we shortlisted Liu et al. [17], as previously stated, it is possible that this study will not be considered for clinical trials by national pharmaceutical regulatory agencies in certain countries. This is because the Lewis lung cancer cells used in the study are derived from mice, not humans. It is also possible for the agency to request additional testing using human-related lung cancer cells to conduct a head-to-head comparison of the pre-clinical outcomes of human and mouse-derived lung cancer cells before approval is given for testing on humans. The liver cancer (Wang et al. [33]) study is the best work to proceed with for a clinical trial due to the presence of sorafenib (a chemotherapy drug) in the PYR group, where the effect of PYR alone on liver cancer in a mouse model can be compared with sorafenib + PYR and control groups.

## Data Availability

All data are available online.

## Code availability

The software name is mentioned in the manuscript
